# Enhancing solar-thermal energy conversion with silicon-cored tungsten nanowire selective metamaterial absorbers

**DOI:** 10.1016/j.isci.2020.101899

**Published:** 2020-12-07

**Authors:** Jui-Yung Chang, Sydney Taylor, Ryan McBurney, Xiaoyan Ying, Ganesh Allu, Yu-Bin Chen, Liping Wang

**Affiliations:** 1 School for Engineering of Matter, Transport & Energy, Arizona State University, Tempe, AZ 85287, USA; 2 Department of Mechanical Engineering, National Chiao Tung University, Hsinchu 300, Taiwan; 3 Department of Power Mechanical Engineering, National Tsing Hua University, Hsinchu 300, Taiwan; 4 Department of Mechanical Engineering, National Cheng Kung University, Tainan 70101, Taiwan

**Keywords:** Thermal Engineering, Thermal Property, Nanomaterials, Energy Materials

## Abstract

This work experimentally studies a silicon-cored tungsten nanowire selective metamaterial absorber to enhance solar-thermal energy harvesting. After conformally coating a thin tungsten layer about 40 nm thick, the metamaterial absorber exhibits almost the same total solar absorptance of 0.85 as the bare silicon nanowire stamp but with greatly reduced total emittance down to 0.18 for suppressing the infrared emission heat loss. The silicon-cored tungsten nanowire absorber achieves an experimental solar-thermal efficiency of 41% at 203°C during the laboratory-scale test with a stagnation temperature of 273°C under 6.3 suns. Without parasitic radiative losses from side and bottom surfaces, it is projected to reach 74% efficiency at the same temperature of 203°C with a stagnation temperature of 430°C for practical application, greatly outperforming the silicon nanowire and black absorbers. The results would facilitate the development of metamaterial selective absorbers at low cost for highly efficient solar-thermal energy systems.

## Highlights

• Si-cored tungsten nanowires are fabricated by conformal coating of 40-nm tungsten
• Total solar absorptance of 0.85 with greatly reduced total emittance is achieved
• Solar-thermal efficiency of 41% at 203°C under 6.3 suns is measured
• 74% efficiency at 203°C with a stagnation temperature of 430°C is projected

## Graphical Abstract

**Figure:**
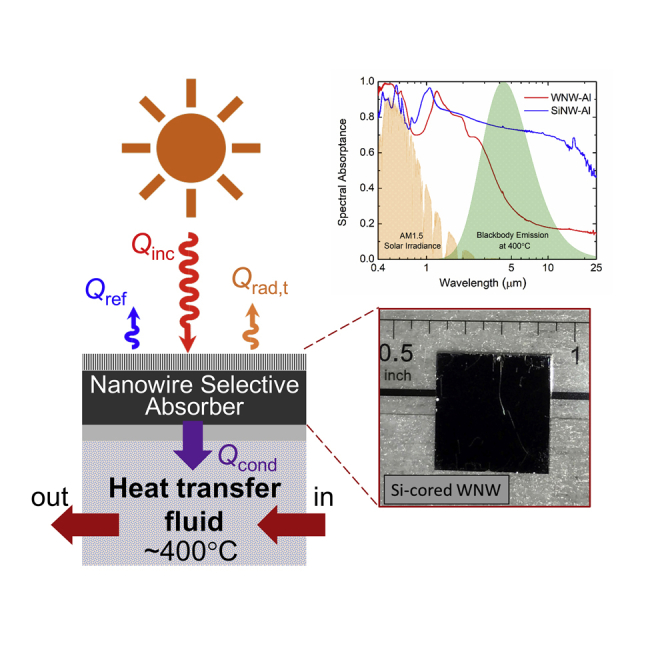

## Introduction

Within the last decade, nanostructured metamaterials have become an attractive topic in the field of radiative heat transfer for thermal energy harvesting (Bello and Shanmugan, 2020; Jin et al., 2016; Khodasevych et al., 2015; Woolf et al., 2018; Xu et al., 2020) and radiative cooling (Raman et al., 2014; Zhai et al., 2017). For harvesting solar energy to heat, spectrally selective absorbers with high solar absorption and low infrared emission are highly desired for efficient energy conversion, and many metamaterial selective solar absorbers have been designed and experimentally demonstrated recently based on multilayer (Chirumamilla et al., 2016, 2019; Dyachenko et al., 2016; Khoza et al., 2019; Thomas et al., 2017; Wang et al., 2018), periodic tungsten convex or concave gratings (Jae Lee et al., 2014; Wang et al., 2015; Wang and Wang, 2013), nickel nanopyramids and tungsten nanowires (WNWs)/doughnuts (Behera and Joseph, 2017; Li et al., 2015; Tian et al., 2018), and nanoporous or nanoparticle composite structures (Lu et al., 2016, 2017; Prasad et al., 2018). Due to their submicron feature sizes, advanced fabrication techniques such as electron-beam lithography and focused-ion beam were usually needed for fabricating these metamaterial structures (Wang et al., 2015), which are expensive with low throughput prohibiting their large-area application. Some cost-effective methods such as direct printing (Han et al., 2016) and interference lithography (Li et al., 2015) in combination with additional thin film deposition and selective etching processes were also successfully demonstrated for fabricating centimeter-scale selective metamaterial absorbers. Simpler and more cost-effective methods are still in need for large-area manufacturing of selective metamaterial solar absorbers. All these experimentally demonstrated metamaterial solar absorbers are achieved via typical periodicity and feature size around a few hundred nanometers, which can be feasibly fabricated with deep UV projection or stepper photolithography (Enrico et al., 2019; Greiner et al., 2006; Park and Lee, 2016). In fact, diffraction gratings for optical devices and nanoimprinting stamps, made of nanopatterned silicon wafers fabricated by deep UV projection lithography along with selective chemical or plasma etching (Opachich et al., 2015; Sanjay et al., 2014), have been commercialized in large area with controllable shape and geometries (Lightsmyth, n.d.).

Moreover, most of reported studies only focused on the fabrication and characterization of the optical and radiative thermal properties to demonstrate the spectral selectivity of metamaterial solar absorbers with the theoretical prediction of their solar-thermal performances. To bridge the gap between material-level studies and device applications, laboratory-scale solar-thermal characterization must be implemented to evaluate the energy conversion performance of developed metamaterial absorbers under different solar concentrations and experimentally ensure the expected performance before large-area manufacturing and more comprehensive outdoor field tests. From an outdoor field test under unconcentrated solar irradiation, a peak temperature of 225°C for a semiconductor-based multilayer selective solar absorber was experimentally obtained inside a vacuum chamber with a glass lid (Thomas et al., 2017). Laboratory-scale photo-thermal conversion experiment with 1-sun illumination was conducted for a Cr grating structure that reached a high temperature of 80.7°C (Seo et al., 2019). A novel laboratory-scale solar-thermal test platform with a broadband solar simulator was successfully developed to characterize the performance of a fabricated metafilm selective solar absorber under multiple solar concentrations up to 20 suns along with detailed heat transfer analysis (Alshehri et al., 2020). So far, systematic laboratory-scale solar-thermal characterization of nanostructured metamaterial selective solar absorbers under variable solar irradiation is still rarely demonstrated.

In this study, we present the simple fabrication, optical characterization, and laboratory-scale solar-thermal tests of a selective metamaterial solar absorber made of silicon-cored WNWs to experimentally demonstrate the improved performance in converting solar energy to heat under multiple solar concentrations due to its excellent spectral selectivity. The fabrication processes will be discussed first along with morphology characterizations, followed by the spectroscopic measurements of the optical and radiative properties in the broad spectrum from solar to infrared. Numerical simulation is then conducted to elucidate the physical mechanisms of high solar absorption and low infrared emission. Last, laboratory-scale solar-thermal tests under different suns are carried out to evaluate and demonstrate its superior conversion performance when compared with the bare silicon nanowire (SiNW) and black absorbers, which is validated by detailed heat transfer analysis.

## Results and discussion

### Sample fabrication and characterization

**Figure 1:**
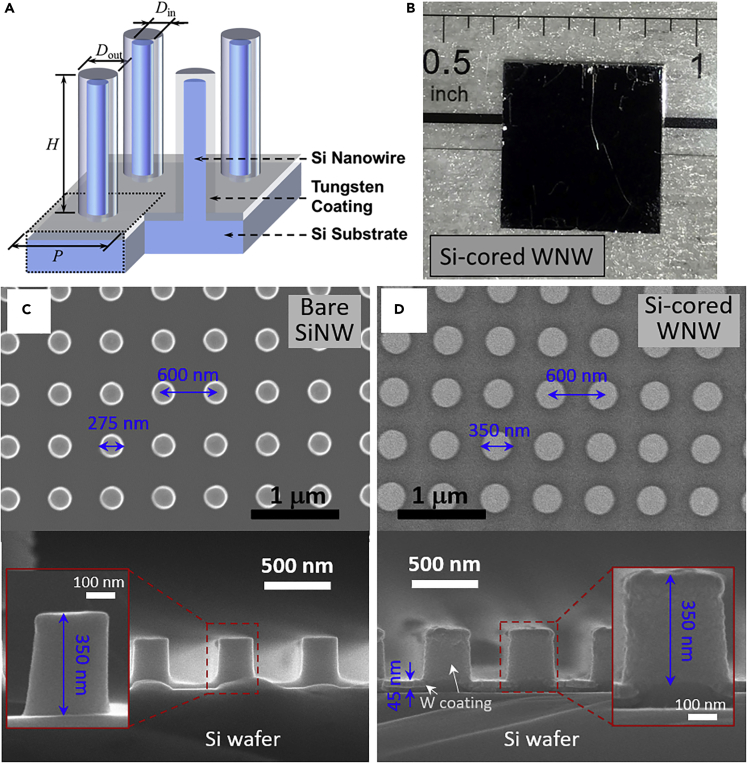
Schematic, photograph, and SEM images (A) Structural schematic of Si-cored tungsten nanowire (WNW) metamaterial as a selective solar absorber where a layer of tungsten is conformally coated on the silicon nanowire (SiNW) surfaces. (B) Photograph of the fabricated Si-cored WNW metamaterial sample in a size of 8 × 8 mm^2^ with a visibly black appearance. (C) Top-view and cross-sectional SEM images of bare SiNW array with nanowire period 600 nm, height 375 nm, and diameter 275 nm without tungsten coating. (D) Top-view and cross-sectional SEM images of Si-cored WNW array with the same period 600 nm, same height 350 nm, and increased nanowire diameter 350 nm after about 40-nm-thick tungsten is conformally sputtered on the silicon surfaces.

The silicon-cored WNW selective metamaterial absorber was fabricated by conformally sputtering a thin tungsten layer (Lesker PVD75) at a deposition rate of 1 Å/s onto a commercially available 2D SiNW stamp (LightSmyth Technologies, S2D-18B2-0808-350-P, 1–10 Ω·cm resistivity, single-side polished, 8 × 8 mm^2^, 675 μm thickness), as illustrated in Figure 1A. Tungsten was selected to be the coating material because of its excellent high-temperature stability in vacuum, high absorption in the visible and near-infrared, and very low emission in the mid-infrared regimen. The fabricated silicon-cored WNW sample is visibly black as seen from the photograph in Figure 1B. Note that another identical sample of the bare SiNW nanostamp without the tungsten coating was used along with the fabricated Si-cored WNW metamaterial sample as solar absorbers for the comparison studies of the spectral radiative properties and solar-thermal tests, which are to be discussed in following sections. A scanning electron microscope (SEM) was used to characterize the geometry of the SiNW and WNW samples as, respectively, shown in Figures 1C and 1D. From the top-view SEM images, it is clearly seen that the period of the nanowires is the same P = 600 nm for both bare SiNW and Si-cored WNW samples before and after the tungsten deposition. The nanowire diameter increases from *D*_in_ = 275 nm to *D*_out_ = 350 nm, indicating that a layer of tungsten about 37.5 nm thick is coated onto the sidewall of SiNWs. It is observed from the cross-sectional SEM images that a continuous tungsten layer was uniformly deposited on the top and side surfaces of SiNWs, which preserves the good cylindrical nanowire shape. The height of the WNW is the same as the SiNW with *H* = 350 nm, thanks to the tungsten layer deposited on the top of nanowire and on the bare silicon substrate inside the grooves with a slightly larger thickness of 45 nm than the sidewalls. Overall, the SEM images confirm the conformal tungsten coating about 40 nm thick from the sputtering process in successfully fabricating Si-cored WNW metamaterial selective absorbers. To ensure opacity, 200-nm-thick aluminum was later sputtered onto the backsides of the samples (denoted as WNW-Al and SiNW-Al) at 1 Å/s.

### Spectral and total radiative properties

**Figure 2:**
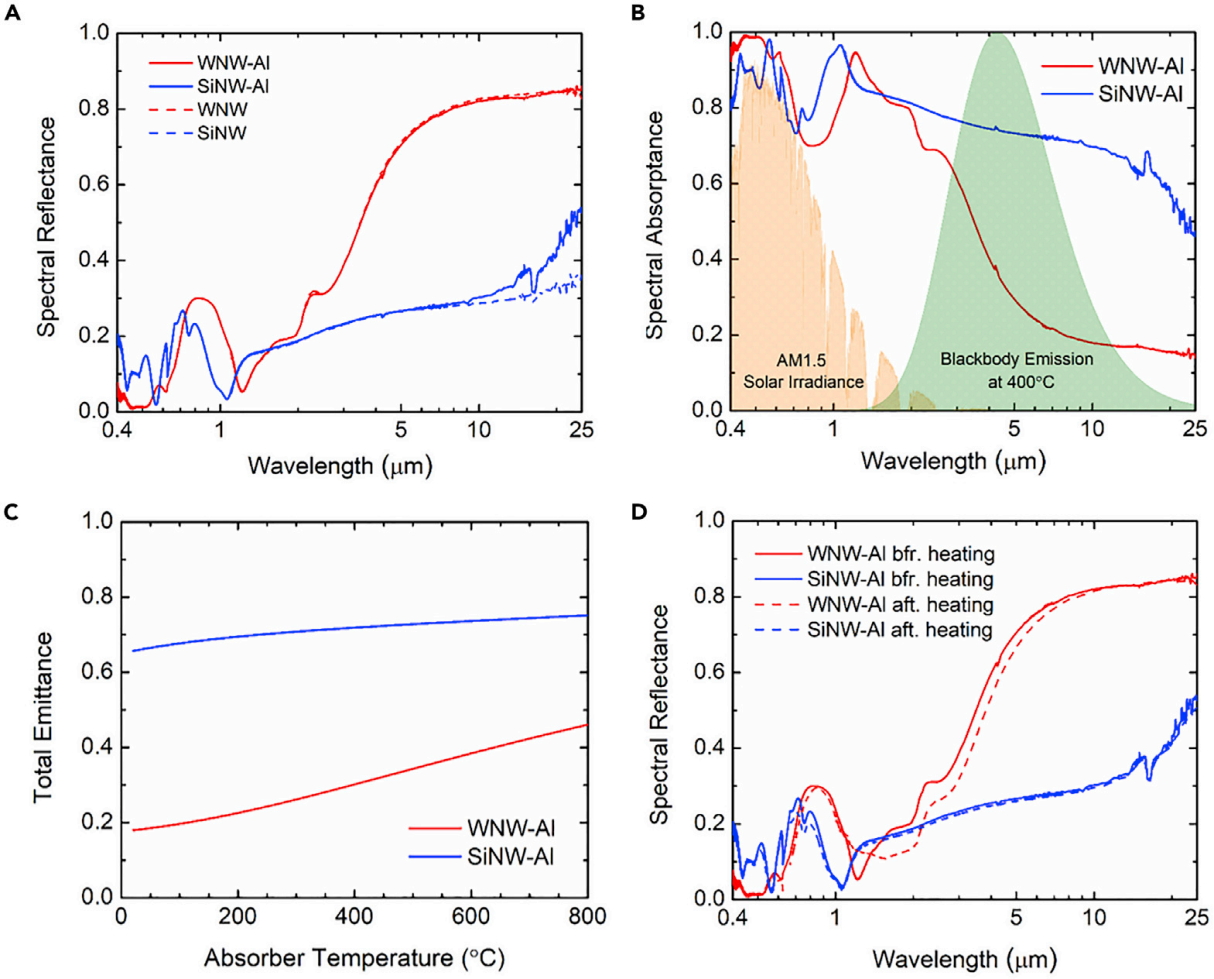
Spectral reflectance, absorptance, and total emittance (A) Measured spectral (near-normal) reflectance (*R*_λ_) in the broad spectral range from 0.4 to 25 μm for bare silicon nanowire (SiNW) and Si-cored tungsten nanowire (WNW) before and after the deposition of 200-nm-thick Al on the backside of samples. (B) Spectral absorptance (or spectral emittance *ε*_λ_ = *α*_λ_ according to Kirchhoff's law) obtained from measured spectral reflectance (i.e., *α*_λ_ = 1−*R*_λ_) in the broad spectral range from visible to mid-infrared for opaque SiNW and Si-cored WNW with Al backside coating (denoted as SiNW-Al and WNW-Al). The shaded areas represent the spectral solar irradiance from air mass 1.5 Global tilt (orange color) and spectral blackbody emissive power at 400°C (green color) both normalized to the peak spectral emissive power of 0.178 W/cm^2^·μm. (C) Calculated total emittance of WNW-Al and SiNW-Al as a function of absorber temperature. (D) Measured spectral reflectance of WNW-Al and SiNW-Al samples in the broad spectral range at room temperature before and after the solar-thermal heating tests in vacuum with a maximum temperature of 375°C for 10 h.

Figure 2A presents the measured spectral (near-normal) reflectance (*R*_λ_) of the SiNW and silicon-cored WNW samples in the broad wavelength range 0.4–25 μm. Experimental details about optical measurements can be found in Supplemental Information. Within the visible and near-infrared, both SiNW and WNW samples exhibit comparable low reflectance with a peak value around 0.30. However, the reflectance of the WNW sample increases abruptly at wavelengths beyond 2 μm, reaching *R*_λ_ = 0.75 around *λ* = 5 μm, whereas at *λ* = 5 μm the bare SiNW sample has a reflectance of 0.30. Note that for a selective solar absorber, the infrared reflectance should be as high as possible to minimize thermal emission losses. The significantly higher infrared reflectance of the WNW sample indicates potentially better solar-thermal conversion than the bare SiNW, thanks to the highly reflective tungsten coating in the infrared. In practical applications, the solar absorber is expected to be opaque such that the thermal emission from the hot heat transfer fluids underneath will not penetrate to lose heat. In fact, the spectral (normal) transmittance measurement reveals that the bare SiNW sample still has about up to 15% transmission from 1 to 3.5 μm in the near-infrared because of the low silicon doping, whereas the WNW sample along with the 40-nm-thick bare tungsten film deposited on lightly doped silicon wafer show only about 2% transmission in the same wavelengths (Figure S1A in the Supplemental Information). Due to the bandgap absorption and large thickness of silicon wafer, the transmission at wavelengths *λ* < 1 μm for both WNW and SiNW samples is effectively zero. Therefore, a 200-nm-thickness Al film was sputtered at a rate of 1 Å/s onto the backsides of both samples to ensure complete opaqueness, which was confirmed by the zero transmittance from the measurement. The broadband reflectance measurement further verifies the effectiveness of the Al backside coating, in particular for bare SiNW, whose infrared reflectance was increased from 0.35 to 0.55 at the wavelength *λ* = 25 μm. On the other hand, the measured spectral reflectance of the WNW barely changes after Al backside coating within the entire spectrum of interest and remains as high as 0.85 in the infrared.

With the Al backside coating, it is reasonable to calculate the spectral absorptance simply by *α*_λ_ = 1−*R*_λ_ and the spectral emittance *ε*_λ_ = *α*_λ_ according to Kirchhoff's law. Note that in principle the spectral reflectance should be hemispherical when calculating the absorptance from energy balance. However, for periodic structures under normal incidence, only specular reflection occurs at wavelengths longer than its period with higher diffraction orders to be evanescent according to the Bloch-Floquet equation for gratings (Wang et al., 2015; Zhang, 2007). Therefore, the measured spectral specular reflectance can be directly used for calculating the spectral normal absorptance for the spectrum with wavelengths *λ* > 0.6 μm for both WNW-Al and SiNW-Al absorbers, whereas it is approximated as the hemispherical reflectance for simplicity here at the shorter spectrum 0.4 μm < *λ* < 0.6 μm.

Figure 2B plots the spectral (normal) absorptance *α*_λ_ (or emittance *ε*_λ_) for both SiNW-Al and Si-cored WNW-Al absorbers, calculated from the spectral reflectance measurements for wavelengths from 0.4 to 25 μm, where the spectral solar irradiance *G*_*λ*_,_AM1.5_ from air mass 1.5 Global tilt (shaded orange) (Air Mass 1.5 Spectra, n.d.) and spectral blackbody emissive power *E*_b_,_*λ*_(*T*) at *T* = 400°C (shaded green) (Modest, 2013), both normalized to the peak spectral emissive power of 0.178 W/cm^2^/μm, are also shown. Clearly the WNW-Al sample has absorption higher than 70% within the entire solar spectrum thanks to several absorption peaks observed at *λ* = 2.44 μm, 1.89 μm, 1.28 μm, and 0.60 μm, whereas its emittance is as low as 0.15 in the infrared, suggesting strong solar absorption and excellent suppression of infrared thermal emission. The numerically modeled spectral absorptance of the WNW-Al absorber verified the measurement (Figure S1B in the Supplemental Information), showing similar low emittance in the infrared down to 0.05 at *λ* = 25 μm. This is possibly due to the theoretical material properties of pure tungsten and similar high absorption within solar spectrum with multiple peaks, whose mechanisms are to be explained next. On the other hand, the SiNW-Al sample has comparably high absorptance in the solar spectrum with major peaks at *λ* = 1.1 μm and 0.60 μm, but its infrared spectral emittance is greater than 0.6 at wavelengths *λ* < 10 μm and is still as high as 0.45 at *λ* = 25 μm, indicating higher thermal emission loss than the WNW sample. Note that the minor absorption peak around *λ* = 15 μm is due to intrinsic optical phonon absorption of silicon (Smith et al., 1985). Undoubtedly the 40-nm tungsten front coating significantly reduced the spectral emittance of the bare SiNW by as much as 0.52 in the wavelength range 3 μm < *λ* < 10 μm where the most of blackbody emission spectrum at 400°C is located, and therefore excellent spectral selectivity is experimentally demonstrated with the silicon-cored WNW absorber.

Note that a black surface at 400°C dissipates heat in thermal emission (i.e., 11.6 kW/m^2^ from $E_{b}=\sigma T^{4}$ ) about 11.6 times the total solar irradiance (i.e., *G*_AM1.5_ = 1 kW/m^2^) without concentration. Therefore, it is crucial to lower the infrared emittance to minimize the heat loss from thermal emission for highly efficient solar-thermal energy harvesting. For better quantitative comparison, the total solar absorptance $\alpha_{solar}$ is, respectively, 0.852 and 0.855 for the WNW-Al and SiNW-Al absorbers, calculated from measured spectral absorptance *α*_*λ*_ and spectral solar irradiation data $G_{\lambda ,AM1.5}$ based on the following equation:

(Equation 1) $$\alpha_{solar}=\frac{\int_{0.4\;\mu m}^{4\;\mu m}\alpha_{\lambda }G_{\lambda ,AM1.5}d\lambda }{\int_{0.4\;\mu m}^{4\;\mu m}G_{\lambda ,AM1.5}d\lambda }$$

On the other hand, the total emittance $\varepsilon_{th}\left(T\right)\;$was calculated with the measured spectral emittance *ε*_*λ*_ and spectral blackbody emissive power *E*_b,*λ*_(*T*) as

(Equation 2) $$\varepsilon_{th}\left(T\right)=\frac{\int_{0.4\;\mu m}^{25\;\mu m}\varepsilon_{\lambda }E_{b,\lambda }\left(\;T\right)d\lambda }{\int_{0.4\;\mu m}^{25\;\mu m}E_{b,\lambda }\left(T\right)d\lambda }$$

As presented in Figure 2C, the total emittance change from room temperature 20°C–400°C is only 0.18 to 0.30 for the WNW-Al absorber but 0.66 to 0.72 for the SiNW-Al absorber. This clearly indicates a significant reduction of 0.42 in total emittance at 400°C by simply coating 40-nm tungsten onto the commercial SiNW nanostamp to achieve excellent spectral selectivity to greatly suppress infrared emission loss while maintaining high solar absorption greater than 85%. When the absorber temperature keeps going up, the total emittance of the WNW-Al absorber increases slightly to 0.38 at 600°C and 0.46 at 800°C, as the blackbody spectrum shifts toward shorter wavelengths and starts to overlap with part of solar spectrum where the spectral absorptance is higher. On the other hand, the total emittance of the SiNW-Al absorber saturates around 0.75 at higher temperatures due to the lack of spectral selectivity.

As thermal stability is another major concern for selective solar-thermal absorbers to robustly operate at high temperatures, the spectral reflectance of both WNW-Al and SiNW-Al samples were measured after heating in vacuum with a maximum temperature of 375°C for 10 h during the solar-thermal tests, which are to be discussed in detail later. As shown in Figure 2D, the measured spectral reflectance barely changes in the broad spectral range before and after the heating, whereas the WNW-Al absorber exhibits no more than an 8% decrease in reflectance (or increase of absorptance) within the infrared from 1.5 to 10 μm due to the possible oxidation of tungsten with residual oxygen atoms during the solar-thermal tests. This indicates the good thermal stability of both WNW-Al and SiNW-Al metamaterial absorbers in vacuum at medium high temperatures.

### Underlying physics for spectral selectivity

**Figure 3:**
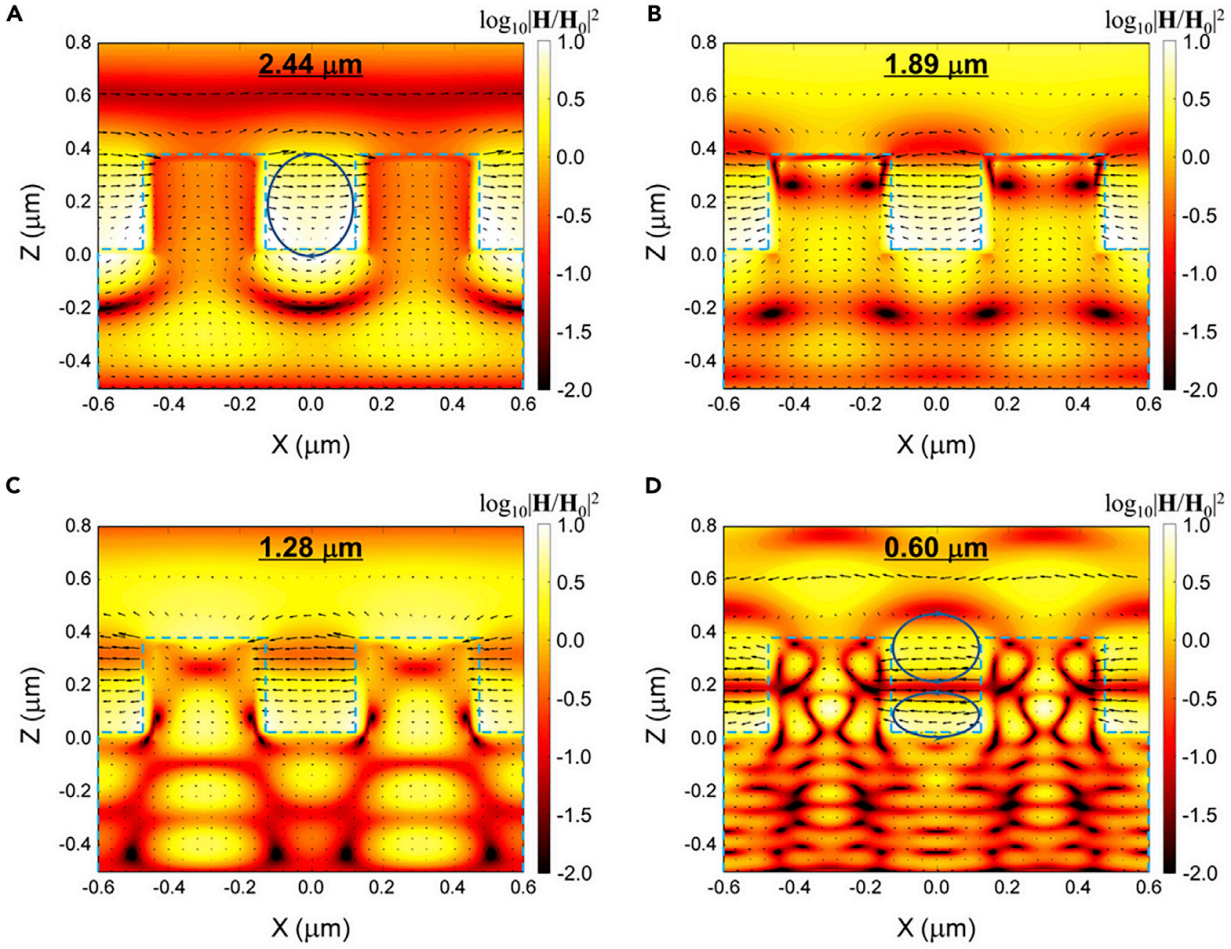
Numerical simulations of electromagnetic field distributions (A–D) For tungsten nanowire arrays with the same geometry (i.e., 600 nm period, 350 nm diameter, 350 nm height) at selected wavelengths of (A) 2.44 μm, (B) 1.89 μm, (C) 1.28 μm, and (D) 0.60 μm where absorptance peaks are observed from the measured spectra. Color contours present the local magnetic field strength, while the arrows indicate the electric field vectors.

To elucidate the physical mechanisms responsible for the high absorption within the solar spectrum and low infrared emission from the WNW-Al absorber, the spectral absorptance was numerically modeled and reasonably agrees with the measured spectrum in Figure S1B. Details for the numerical simulation method can be seen in the Supplemental Information, and the effects of geometric parameters (i.e., nanowire period, diameter, and height) on spectral absorptance of WNW metamaterial absorbers were calculated in Figure S2. Here the simulated electromagnetic field distributions in the x-z cross-section for two consecutive unit cells of the nanowire array are plotted in Figure 3 at the four wavelengths where spectral absorptance peaks are observed. Note that the contour color indicates the magnetic field strength normalized to the incident field and the black arrows represent the electric field vectors. As shown in Figure 3A for the wavelength *λ* = 2.44 μm, a strong magnetic field confinement is located in the vacuum spacing between two neighboring WNWs, whereas the electric field vectors indicate an electric current loop (see Figure S3 for Poynting vector distribution in the Supplemental Information). This can be confirmed as the first harmonic mode of magnetic polariton (MP) as discussed in our previous theoretical study of WNW solar absorbers, which can be further verified by an inductor-capacitor circuit model prediction of 2.375 μm (Chang et al., 2017). For the wavelengths of 1.89 and 1.28 μm, whose electromagnetic fields are, respectively, presented in Figures 3B and 3C, the enhancement is not caused by MP. In fact, without the electrical field forming a loop, the confinement is no longer classified as MP or artificial magnetic response. The main cause of the enhancement peaks is interference inside the substrate as well as the intrinsic absorption of tungsten. However, as the tungsten layer is thick enough to absorb or reflect most of the incident wave in the simulation, the effect of interference within the substrate is not as strong as MP, which confines energy between NWs. That is, these two peaks are not as distinct as MP1 (first harmonic term of MP). On the other hand, the last peak at 0.60 μm is caused by the well-known Wood's anomaly, which can be predicted analytically at 0.59 μm wavelength with the utilization of the diffraction equation for periodic arrays and effective medium theory (Chen et al., 2006; Chen and Tan, 2010; Ho et al., 2016).

### Solar-thermal test and performance analysis

**Figure 4:**
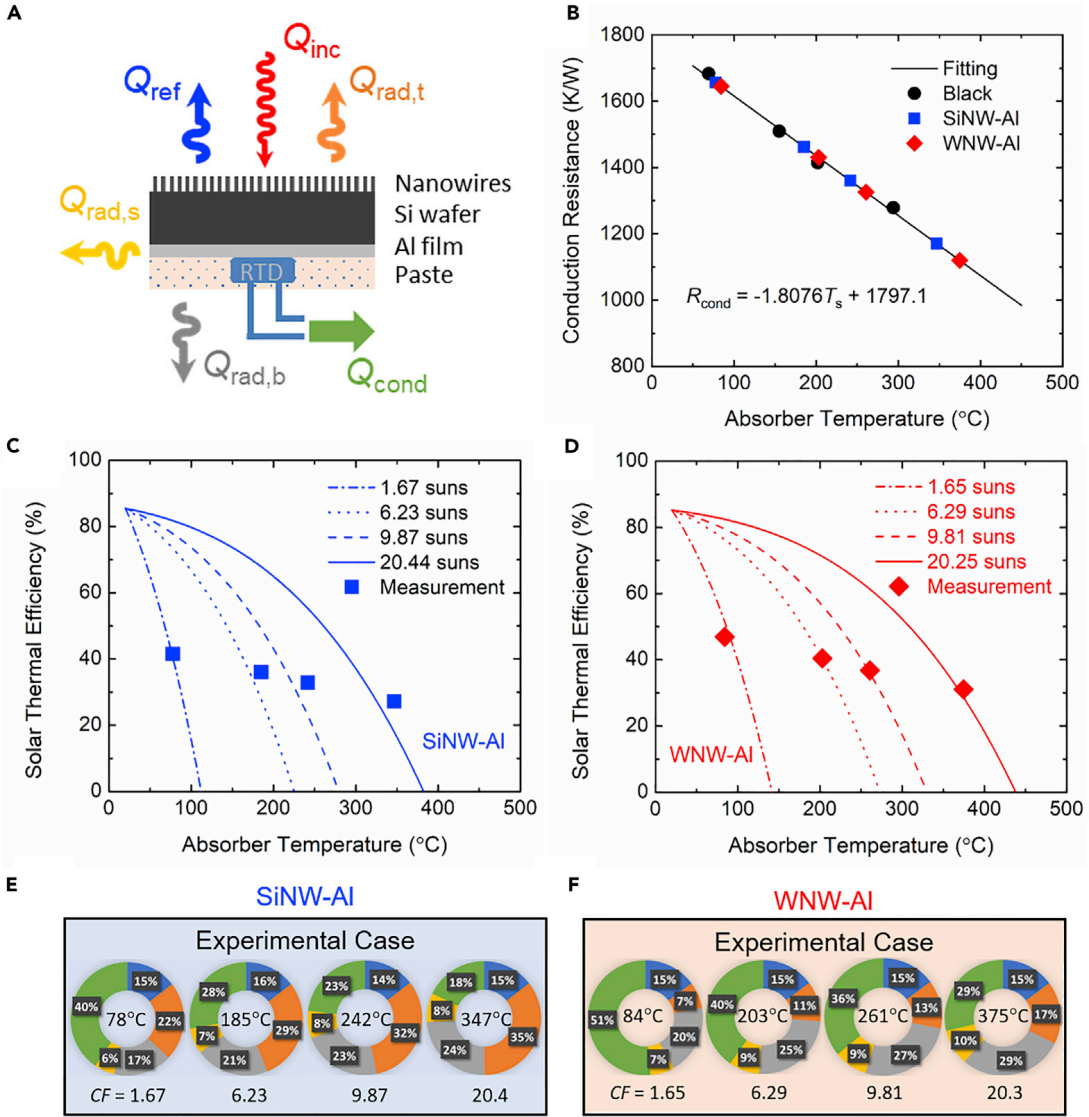
Laboratory-scale solar-thermal test (A) Schematic of heat transfer modes for nanowire absorbers during the laboratory-scale solar-thermal testing at different solar concentrations. Note that the convective heat loss is neglected as the test was conducted under high vacuum, and the absorber temperature was measured by a resistance temperature detector (RTD) attached at the backside with thermal paste. (B) Calibration of conduction resistance for the conduction heat loss via the RTD wires (considered as a useful heat gain) at multiple absorber temperatures with a black absorber, based on which the conduction heat gain is calculated for nanowire samples from the measured temperatures. (C and D) Measured solar-thermal efficiency (markers) at multiple solar concentrations (from ~1.6 to ~20 suns) for fabricated (C) SiNW-Al and (D) WNW-Al absorber samples in good agreement with the theoretical prediction (lines). (E and F) Heat transfer analysis pie charts of energy loss ratios for (E) SiNW-Al and (F) WNW-Al samples at measured absorber temperatures under corresponding solar concentration factors (CF).

To experimentally demonstrate the performance of the WNW-Al and SiNW-Al samples as selective solar absorbers, a laboratory-scale solar-thermal test with a custom-built setup consisting of a 1-kW solar simulator, a 1-ft^3^ vacuum chamber, and optical filters and mirrors was conducted at selected concentrated solar irradiation from 1 up to 20 suns by different combinations of neutral density filters. A detailed description of the solar-thermal test apparatus can be found elsewhere (Alshehri et al., 2020) and thus will not be discussed here. As depicted in Figure 4A, a 2 × 2 mm^2^ resistance temperature detector (RTD, OMEGA, F2020-100-B-100), which measures the absorber temperature up to 500°C, was adhered onto the backside of the nanowire absorber samples by thermal paste, which covered the entire back surface. Three independent tests under the same solar concentration were conducted under a vacuum pressure less than 1 × 10^−3^ Torr achieved by a turbo vacuum pump for the same sample, and the averaged absorber temperature at steady state with a standard deviation of less than 3% was reported here.

With convection eliminated and negligible conduction by air molecules under high vacuum, the energy balance at steady state for the absorber yields

(Equation 3) $$Q_{\text{inc}}=Q_{\text{ref}}+\sum_{i=\text{t},\text{b},\text{s}}Q_{\text{rad},i}+Q_{\text{cond}}$$

Note that $Q_{\text{inc}}\;$ is the incident solar irradiation measured by a power sensor (Thorlabs, S310C) and the solar concentration factor can be calculated as $CF=\;Q_{\text{inc}}/\left(A_{\text{t}}G_{\text{AM}1.5}\right)$. $Q_{\text{ref}}=\left(1-\alpha_{solar}\right)Q_{\text{inc}}$ is the reflected solar irradiation by the front absorber surface, and $Q_{\text{rad},i}=A_{i}\varepsilon_{i}\text{σ}\left(T^{4}-T_{\infty }^{4}\right)$ is the radiated heat loss from top, bottom, and side surfaces (i.e., *i* = t, b, s) to the vacuum chamber wall at *T*_∞_ = 20°C, where $A_{i}$ and $\varepsilon_{i}\;$ are the area and total emittance of corresponding surfaces, respectively. The top surface is the nanowire absorber whose temperature-dependent total emittance is taken from Figure 2C, whereas the side and bottom surfaces are, respectively, tungsten and thermal paste whose temperature-dependent total emittance was found from previous optical measurements (Alshehri et al., 2020). $Q_{\text{cond}}$ is the conducted heat via RTD wires considered as useful heat gain during the solar-thermal test, and the experimental solar-thermal efficiency can be thereby defined as

(Equation 4) $$\eta_{\text{exp}}=\frac{Q_{\text{cond}}}{Q_{\text{inc}}}=\frac{T-T_{\infty }}{R_{\text{cond}}\left(T\right)Q_{\text{inc}}}$$

where *R*_cond_(*T*) is the conduction resistance of the RTD wires that is dependent on the absorber temperature *T*. As $Q_{\text{cond}}$ cannot be directly measured, a commercial black absorber (Acktar, n.d.) with solar absorptance of 0.998 and total emittance around 0.93 characterized previously (Alshehri et al., 2020) was used to calibrate the temperature-dependent conduction heat transfer via the RTD wires. The steady-state temperature of the black absorber was measured during the solar-thermal test for solar concentrations 1.1, 4.0, 6.4, and 13.3 suns (see Figure S4 in the Supplemental Information) for calculating $Q_{\text{cond}}\left(T\right)$ from the energy balance in Equation (3), based on which the conduction resistance *R*_cond_(*T*) was fitted into a linear function of absorber temperature with $R_{\text{cond}}=-1.8076T+1797.1$. As shown in Figure 4B, the experimental conduction resistance *R*_cond_ or conduction heat transfer $Q_{\text{cond}}$ of the WNW-Al and SiNW-Al absorbers can be found from their measured steady-state temperatures during the solar-thermal tests according to the linear relation, from which the experimental solar-thermal efficiency $\eta_{\text{exp}}$ was calculated.

Figures 4C and 4D present the measured solar-thermal efficiency under multiple solar concentrations from the solar-thermal tests for the fabricated SiNW-Al and WNW-Al absorbers, respectively. In particular, with parasitic radiative losses, the WNW-Al absorber achieved experimental solar-thermal efficiencies of 47%, 41%, 37%, and 31% under 1.7, 6.3, 9.8, and 20 suns, which is about 5% (absolute value) higher than that of the SiNW-Al absorber under the same solar concentration. Considering almost the same solar absorptance, this solar-thermal efficiency enhancement directly observed from the solar-thermal test is undoubtedly due to the excellent spectral selectivity of the WNW-Al absorber with a much smaller total emittance that suppressed the thermal emission loss. Note that the SiNW-Al absorber with relatively higher emittance still performs much better than the black absorber by 11% (absolute value) higher experimental efficiency from the solar-thermal test under 6.4 suns. Theoretical efficiencies were also calculated by subtracting the radiative losses from all the surfaces and the reflected solar irradiance from the incidence as

(Equation 5) $$\eta_{\text{theo}}=\frac{Q_{\text{inc}}-Q_{\text{ref}}-\sum_{i=\text{t},\text{b},\text{s}}Q_{\text{rad},i}}{Q_{\text{inc}}}$$

The calculated theoretical efficiencies agree well with the experimentally measured ones with less than 3% difference for both SiNW-Al and WNW-Al absorbers, which validates the solar-thermal test result. In particular, the modeling also suggests that the WNW-Al absorber could reach the stagnation temperatures (the highest temperature with zero efficiency or no heat gain) of 142°C, 273°C, 330°C, and 438°C under 1.67, 6.23, 9.87, and 20.44 suns, which is 30°C–56°C higher than the those of SiNW-Al absorber for the laboratory-scale solar-thermal experiment. As, respectively, shown in Figures 4E and 4F, heat transfer analysis pie charts plot the ratios of each heat transfer mode to the incident solar irradiation for the SiNW-Al and WNW-Al samples at the measured absorber temperatures under different solar concentration factors (CF). Both nanowire absorbers reflect about the same 15% (blue) of incident solar energy, whereas the WNW-Al absorber loses 7%–17% of total energy from its top surface (orange), compared with 22%–35% from the SiNW-Al absorber from 1.67 to 20.4 suns, thanks to the greatly reduced thermal emittance with about 40-nm tungsten coating. On the other hand, during the solar-thermal tests both samples suffer from the parasitic radiative losses from the side (yellow) and bottom (gray) surfaces, which account for 23%–39% of the energy losses.

During practical applications such as parabolic trough systems, the radiative losses from the side or bottom surfaces can be possibly eliminated as the selective solar absorber can be coated on the inner surfaces of evacuated circular tubes, in which case the projected solar-thermal efficiency is

(Equation 6) $$\eta_{\text{proj}}=1-\frac{Q_{\text{ref}}+Q_{\text{rad},\text{top}}}{Q_{\text{inc}}}=\alpha_{solar}-\frac{\varepsilon_{th}\sigma \left(T^{4}-T_{\infty }^{4}\right)}{CF\cdot G_{\text{AM}1.5}}$$

**Figure 5:**
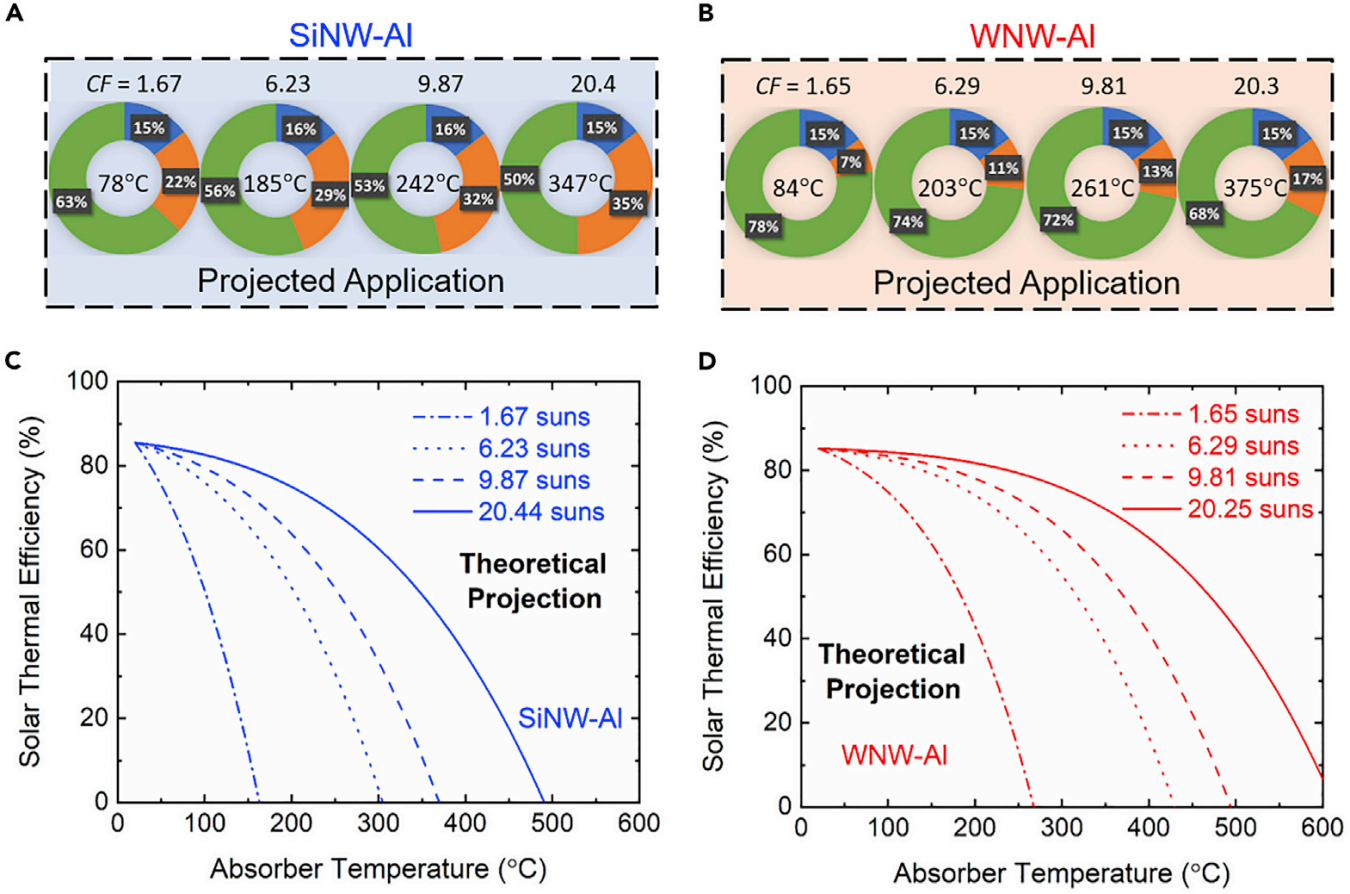
Projected solar-thermal performance (A–D) (A and B) Heat transfer analysis pie charts of the energy loss ratios for SiNW-Al and WNW-Al samples at measured absorber temperatures under corresponding solar concentration factors (CF), and (C and D) theoretically predicted solar-thermal efficiency at multiple solar concentrations for fabricated SiNW-Al and WNW-Al samples as a function of absorber temperature for projected solar-thermal applications where the radiation losses from the side and bottom surfaces can be eliminated.

Figures 5A and 5B show the heat transfer analysis pie charts for the projected applications with both SiNW-Al and WNW-Al absorbers. The percentage of energy from the incident solar radiation that is converted into useful heat gain (green color), i.e., solar-thermal efficiency, is increased to 78%–68% with WNW-Al from 63% to 50% with the SiNW-Al absorber, for solar concentrations from 1.6 to 20 suns at the corresponding temperatures measured from the laboratory-scale solar-thermal tests. Figures 5C and 5D plot the projected solar-thermal efficiency as a function of absorber temperature at multiple solar concentrations for the SiNW-Al and WNW-Al absorbers, respectively. With the eliminated radiative losses from side and bottom surfaces, the efficiency for the projected application and the stagnation temperatures are greatly increased compared with those measured from the laboratory-scale solar-thermal test. In particular, at solar concentration of 6.3 suns, the WNW-Al absorber could reach a stagnation temperature of 430°C, which is 125°C higher than the SiNW-Al absorber, or 146°C higher than the black absorber, by virtue of the excellent spectral selectivity with about 40-nm-thick conformal tungsten coating onto the SiNW sample. Additional calculations also show that the WNW-Al absorber could achieve a stagnation temperature of 238°C under unconcentrated solar irradiation (i.e., *CF* = 1), which is more than 100°C higher than the SiNW-Al or black absorbers, and reach projected solar-thermal efficiency of 51% under the absorber temperature of 400°C at 10 suns, at which neither the SiNW-Al nor black absorber can obtain any net heat input (Figure S5 in the Supplemental Information).

### Conclusion

We experimentally demonstrated enhanced solar-thermal energy conversion with Si-cored WNW selective metamaterial absorber, which was simply fabricated by conformally coating about 40-nm-thick tungsten onto a commercial SiNW stamp along with a 200-nm Al backside coating to ensure the opaqueness. Optical spectroscopic measurements clearly showed the greatly improved spectral selectivity for the WNW-Al absorber with almost the same total solar absorptance of 0.85 and significantly reduced total emittance in the infrared from 0.18 to 0.38 at room temperature up to 600°C. Numerical simulations elucidated the mechanisms of several spectral absorptance peaks within the solar spectrum generated by magnetic resonance, wave interference, intrinsic absorption, and Wood's anomaly. Laboratory-scale solar-thermal tests further confirmed the greatly improved solar-to-heat conversion efficiency with detailed heat transfer analysis for the WNW-Al absorber over the SiNW-Al and black ones at multiple solar concentrations. In particular, the WNW-Al absorber achieved an experimental efficiency of 41% at a temperature of 203°C during the solar-thermal tests with a stagnation temperature of 273°C under 6.3 suns, which are projected to reach 74% efficiency at the same temperature of 203°C with stagnation temperature of 430°C for practical application without parasitic radiative losses from side and bottom surfaces, dramatically outperforming the SiNW-Al and black absorbers. The findings would facilitate the development of novel metamaterial-based selective absorbers at low cost for highly-efficient solar energy harvesting and conversion.

### Limitations of the study

Current laboratory-scale solar-thermal test is limited to temperatures less than 500°C due to the particular RTD sensor used, while it can be possibly improved up to 700°C with a high-temperature thermal sensor. The parasitic radiative losses during the solar-thermal test could be further reduced to achieve higher solar-thermal efficiency. Although the good thermal stability of Si-cored WNW metamaterial absorber was suggested when heating in vacuum at 375°C for 10 h, more comprehensive thermal tests at much longer heating time, higher temperatures, and multiple heating/cooling cycles to imitate the practical scenario are needed to confirm its excellent thermal stability before field applications.

### Resource availability

#### Lead contact

Liping Wang, email liping.wang@asu.edu.

#### Materials availability

Bare silicon nanowire stamps are commerically available from Lightsmyth.

#### Data and code availability

Upon reasonable request from the Lead Contact.

## Methods

All methods can be found in the accompanying Transparent Methods supplemental file.

## Supplemental information

Document S1. Transparent Methods and Figures S1–S5

## Footnotes

Supplemental Information can be found online at https://doi.org/10.1016/j.isci.2020.101899.
