# Supplementary material for: Enhancing solar-thermal energy conversion with silicon-cored tungsten nanowire selective metamaterial absorbers
Source: iScience. 2020 Dec 7;24(1):101899. doi: 10.1016/j.isci.2020.101899 (PMC7753143; doi:10.1016/j.isci.2020.101899)
Supplement: Document S1. Transparent Methods and Figures S1–S5 [file mmc1.pdf]

iScience, Volume *24*

Supplemental Information

Enhancing solar-thermal energy conversion with silicon-cored tungsten nanowire selective metamaterial absorbers

Jui-Yung Chang, Sydney Taylor, Ryan McBurney, Xiaoyan Ying, Ganesh Allu, Yu-Bin Chen, and Liping Wang

Supplemental Information

Enhancing Solar Thermal Energy Conversion with Silicon-cored Tungsten Nanowire Selective Metamaterial Absorbers

Jui-Yung Chang,[1,2,3] Sydney Taylor,[1] Ryan McBurney,[1] Xiaoyan Ying,[1] Ganesh Allu,[2] Yu-Bin Chen,[3,4] and Liping Wang[1,*]

[1] School for Engineering of Matter, Transport & Energy
Arizona State University, Tempe, AZ 85287, USA
[2] Department of Mechanical Engineering
National Chiao Tung University, Hsinchu City 300, Taiwan
[3] Department of Power Mechanical Engineering
National Tsing Hua University, Hsinchu City 300, Taiwan
[4] Department of Mechanical Engineering
National Cheng Kung University, Tainan 70101, Taiwan

[*]Corresponding Author and Lead Contact. Email: liping.wang@asu.edu, Phone: 1-480-727-8615

Transparent Methods

Optical measurements for spectral and total radiative properties

The spectral reflectance of the bare SiNW and silicon-cored WNW samples were characterized by a Fourier-transform spectrometer (Thermo Scientific, Nicolet iS50) along with a 10° specular reflectance accessory (PIKE Technologies, 10Spec) without any polarizer at room temperature. A tungsten-halogen white-light source along with a quartz beamsplitter and a silicon detector was used for the visible to the near-infrared (i.e., 0.4 μm to 1.0 μm), while a Polaris infrared source along with an extended KBr beamsplitter and a DTGS detector was employed for the longer wavelengths from 1 μm to 25 μm. Each spectrum was averaged from 32 scans at a resolution of 2 cm^{-1}, and a freshly deposited 200-nm aluminum mirror was used as the reflection reference. Averaged spectra from at least three independent measurements were reported here after the correction with the theoretical reflectance of aluminum, whose optical constants were taken from Palik tabulated data for the calculation.

Numerical optical simulation

The finite-difference time-domain method was employed to simulate the optical responses of the Si-cored WNW absorber with the same geometry as the fabricated sample in a broad wavelength range from 0.4 μm to 25 μm. The dispersive optical constants of both tungsten and silicon were obtained from Palik's tabular data (Smith et al., 1985). A plane wave with 0° polarization angle was set as the source with normal incidence, at which the 2D nanowire structure is polarization-independent due to in-plane symmetry. Bloch boundary conditions were applied at in-plane directions while perfectly matched layers were used at out-of-plane directions to avoid boundary reflections, which simulated an opaque structure with zero transmittance. Therefore, the spectral absorptance of Si-cored WNW can be calculated by $\alpha_\lambda = 1 - R_\lambda$ where the spectral reflectance R_λ was calculated from the power and field monitors. Non-uniform meshes with a minimum size of 4 nm were used to ensure convergence with a relative error less than 0.5%.

Supplemental Figures

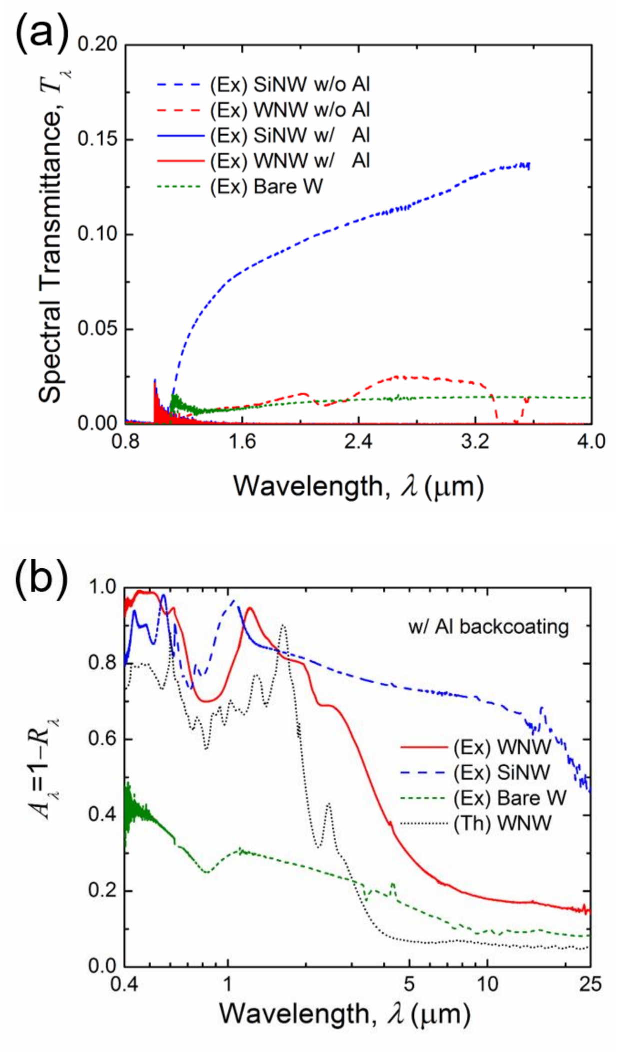

Figure S1 Spectral transmittance and absorptance (Related to Figure 2). (a) Measured near-infrared spectral normal transmittance of bare silicon nanowire (SiNW) and Si-cored tungsten nanowire (WNW) absorbers before and after the deposition of 200-nm-thick Al on their backsides along with a 200-nm-thick bare tungsten film deposited on a silicon wafer. (b) Experimentally measured spectral absorptance of bare tungsten film, SiNW and WNW with Al backside coatings in the broad range from visible to mid-infrared along with the numerically calculated spectral absorptance for tungsten nanowire for comparison.

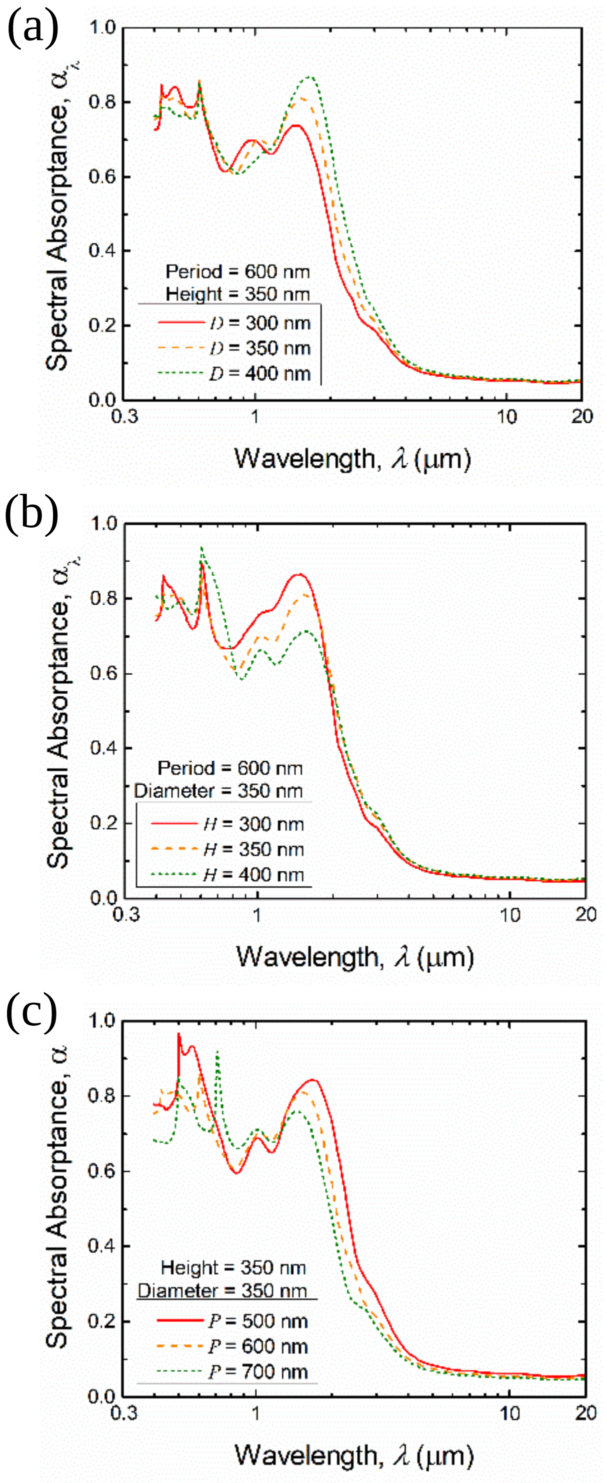

Figure S2 Simulated geometric effects on spectral absorptance (Related to Figure 2). Numerically simulated spectral absorptance of tungsten nanowire (WNW) absorbers when one geometric factor is varied: (a) nanowire diameter *D*, (b) nanowire height *H*, and (c) nanowire period *P*. The geometry from the manuscript (P = 600 nm, H = 350 nm, D = 350 nm) is used as the base one.

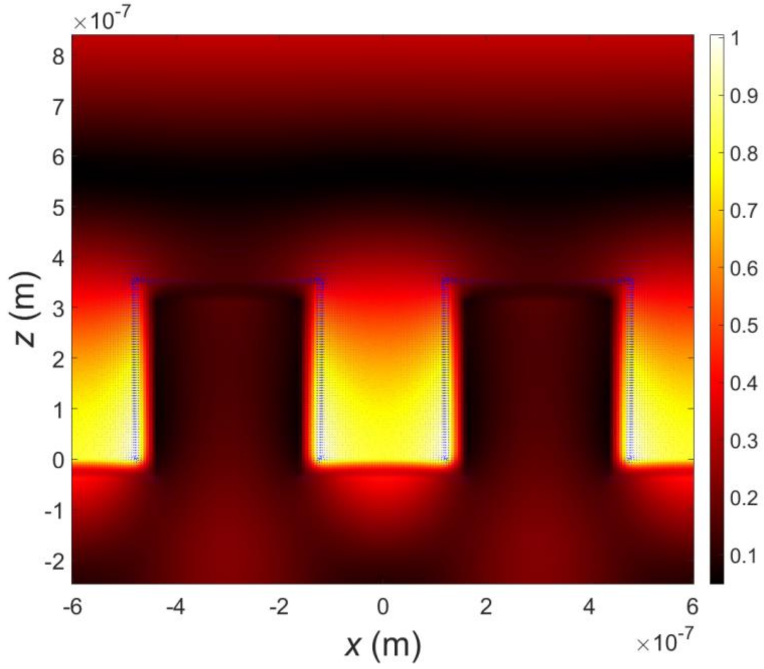

Figure S3 Numerically simulated time-averaged Poynting vectors (Related to Figure 3). Normalized to the incidence inside the WNW absorber with the base geometry (*P* = 600 nm, *H* = 350 nm, *D* = 350 nm) at the wavelength λ = 2443 nm.

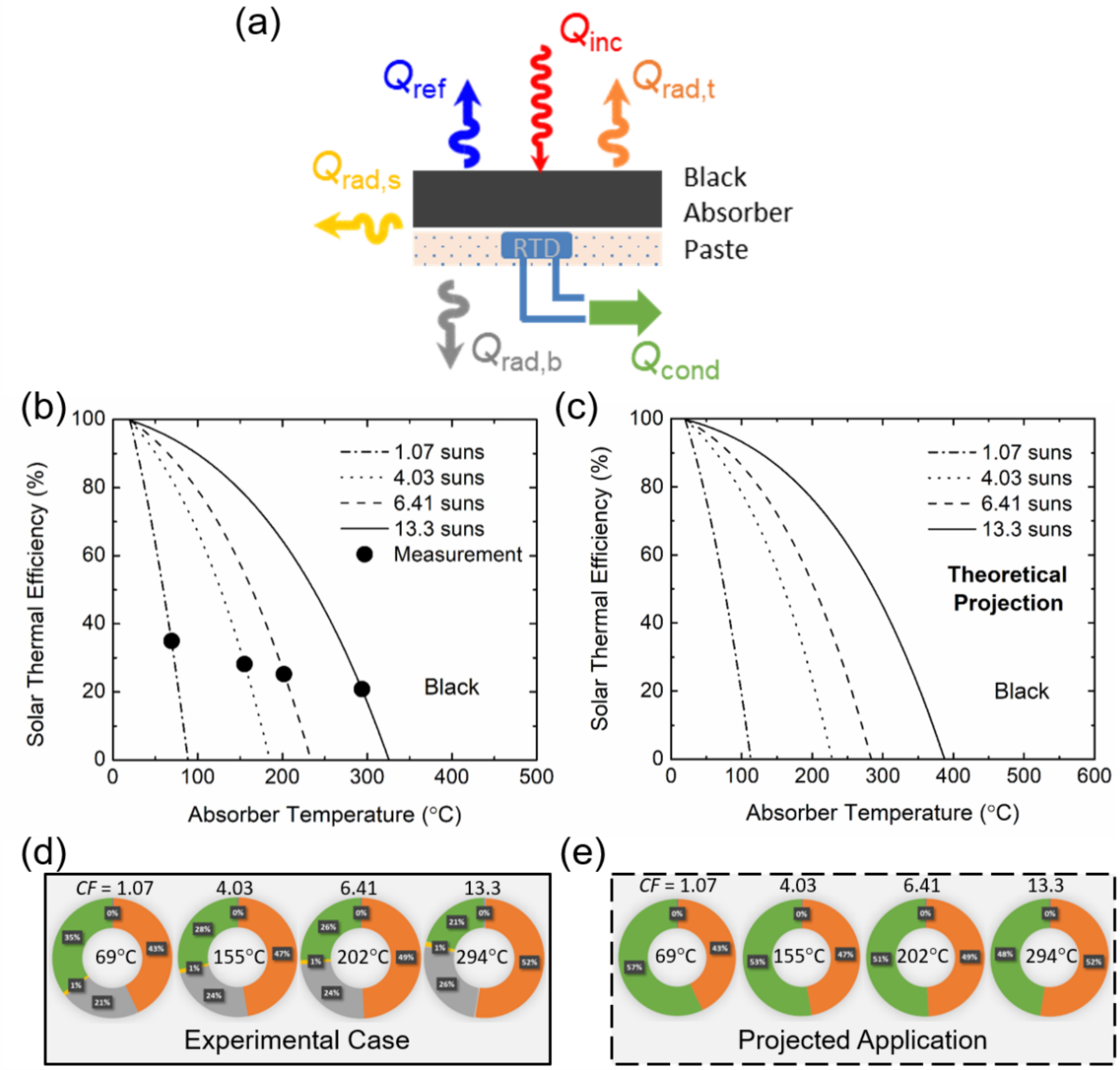

Figure S4 Solar-thermal performance of the black absorber (Related to Figure 4 and 5). (a) Schematic of heat transfer modes for black absorber (Actar) during the lab-scale solar thermal testing at different solar concentrations. Note that the convective heat loss is neglected as the test was conducted under high vacuum, and the absorber temperature was measured by a resistance temperature detector (RTD) adhered at the backside with thermal paste. (b) Measured solar thermal efficiency (markers) at multiple solar concentrations (from ~1 to ~13 suns) for the black absorber (Actar) along with the theoretical prediction (lines) for the experimental case. (c) Theoretically predicted solar thermal efficiency at multiple solar concentrations for the black absorber (Actar) as a function of absorber temperature for projected solar thermal applications where the radiation losses from the side and bottom surfaces can be eliminated. (d-e) Heat transfer analysis pie charts for illustrating the energy loss ratios for the black absorber (Actar) at measured absorber temperatures under corresponding solar concentration factors (CF) for the experimental case and the projected application.

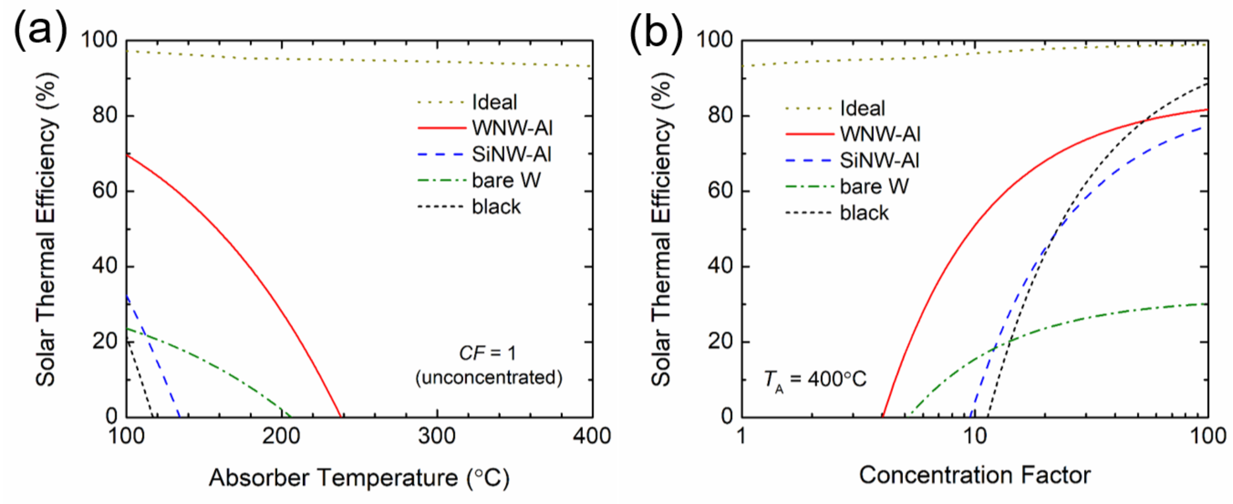

Figure S5 Predicted solar thermal conversion efficiency (Related to Figure 5). The comparison of predicted solar thermal conversion efficiency among Si-cored tungsten nanowire (WNW-Al), bare silicon nanowire with Al backside coating (SiNW-Al), bare 200-nm-thick tungsten on silicon wafer, the black (Actar) and ideal absorber (a) as a function of absorber temperature under 1 sun (unconcentrated solar) and (b) as a function of concentration factor at fixed absorber temperature of 400°C. Note that the experimentally measured spectral absorptance in the broad spectral range from 0.4 μm to 25 μm at room temperature were used in the calculations for all the absorber samples except for the ideal one which has 100% absorptance below and zero emittance above the cutoff wavelength of 1.8 μm.
